# General anesthesia vs. non-general anesthesia for vertebrobasilar stroke endovascular therapy

**DOI:** 10.3389/fneur.2023.1104487

**Published:** 2023-02-02

**Authors:** Yanan Lu, Pengfei Xu, Jinjing Wang, Lulu Xiao, Pan Zhang, Zuowei Duan, Dezhi Liu, Chaolai Liu, Delong Wang, Di Wang, Chao Zhang, Tao Yao, Wen Sun, Zhaozhao Cheng, Min Li

**Affiliations:** ^1^Stroke Center and Department of Neurology, The First Affiliated Hospital of USTC, Division of Life Sciences and Medicine, University of Science and Technology of China, Hefei, Anhui, China; ^2^Department of Neurology, Affiliated Jinling Hospital, Medical School of Nanjing University, Nanjing, Jiangsu, China; ^3^Department of Neurology, Second Affiliated Hospital of Xuzhou Medical University, Xuzhou, Jiangsu, China; ^4^Department of Neurology, Shuguang Hospital Affiliated With Shanghai University of Traditional Chinese Medicine, Shanghai, China; ^5^Department of Neurology, The First People's Hospital of Jining, Jining, Shandong, China; ^6^Department of Anesthesiology, The First Affiliated Hospital of USTC, Division of Life Sciences and Medicine, University of Science and Technology of China, Hefei, Anhui, China; ^7^Department of Critical Care Medicine, The First Affiliated Hospital of USTC, Division of Life Sciences and Medicine, University of Science and Technology of China, Hefei, Anhui, China; ^8^Department of Neurology, Jiangsu Province Hospital of Chinese Medicine, Affiliated Hospital of Nanjing University of Chinese Medicine, Nanjing, China

**Keywords:** vertebrobasilar occlusion, anesthesia, endovascular treatment, outcome, propensity score

## Abstract

**Background:**

The optimal type of anesthesia for acute vertebrobasilar artery occlusion (VBAO) remains controversial. We aimed to assess the influence of anesthetic management on the outcomes in VBAO patients received endovascular treatment (EVT).

**Methods:**

Patients underwent EVT for acute VBAO at 21 stroke centers in China were retrospectively enrolled and compared between the general anesthesia (GA) group and non-GA group. The primary outcome was the favorable outcome, defined as a modified Rankin Scale (mRS) score 0–3 at 90 days. Secondary outcomes included functional independence (90-day mRS score 0–2), and the rate of successful reperfusion. The safety outcomes included all-cause mortality at 90 days, the occurrence of any procedural complication, and the rate of symptomatic intracranial hemorrhage (sICH). In addition, we performed analyses of the outcomes in subgroups that were defined by Glasgow Coma Scale (GCS) score (≤8 or >8).

**Results:**

In the propensity score matched cohort, there were no difference in the primary outcome, secondary outcomes and safety outcomes between the two groups. Among patients with a GCS score of 8 or less, the proportion of successful reperfusion was significantly higher in the GA group than the non-GA group (aOR, 3.57, 95% CI 1.06–12.50, *p* = 0.04). In the inverse probability of treatment weighting-propensity score-adjusted cohort, similar results were found.

**Conclusions:**

Patients placed under GA during EVT for VBAO appear to be as effective and safe as non-GA. Furthermore, GA might yield better successful reperfusion for worse presenting GCS score (≤8).

**Registration:**

URL: http://www.chictr.org.cn/; Unique identifier: ChiCTR2000033211.

## Introduction

Despite remarkable advances in the endovascular treatment (EVT) of large artery occlusion in acute ischemic stroke, the clinical outcomes have not kept pace. Among the numerous studies of the reasons for this mismatch, perioperative management has received comparatively little attention in terms of affecting clinical outcomes. Prior observational studies have suggested that patients undergoing EVT without general anesthesia (non-GA) have a higher probability of good clinical outcomes than patients treated with general anesthesia (GA) (1, 2). Non-GA may lead to faster initiation of therapy and may avoid complications associated with intubation, however, the detrimental effect of GA was ultimately mediated through infarct growth (3). The well-known randomized trials (GOLIATH, SIESTA, and ANSTROKE) compared general anesthesia to conscious sedation (CS) during EVT, but the conclusions were inconsistent (4–6).

To our knowledge, most observational studies and prospective randomized controlled trials (RCTs) have been limited to enrolling patients with anterior circulation stroke (3, 4, 6, 7). Few studies have been conducted on the types of anesthetics that may impact functional outcomes in acute vertebrobasilar occlusion (VBAO) patients treated with EVT (8, 9). Unlike anterior circulation strokes, a considerable proportion of patients with posterior circulation strokes require emergency intubation for airway protection due to alterations in the level of consciousness. For patients with poorer clinical presentation and more severe stroke, it is worth exploring whether GA is more beneficial than non-GA. Therefore, the best anesthetic management for VBAO is still unclear.

We aimed to determine whether the use of GA for EVT of VBAO was safe and to compare the differences in clinical outcomes between GA and non-GA based on acute PostErior ciRculation iSchemIc Stroke regisTry (PERSIST), a retrospective multicenter EVT registry program of VBAO treated with EVT in China.

## Methods

Data from this study are available from the corresponding author upon reasonable request.

### Study population

The retrospective PERSIST recruited stroke centers within China to submit demographic, clinical presentation, procedural details, angiographic and clinical outcome data on consecutive patients who present with acute, symptomatic, radiologically verified VBAO treated with EVT at 21 stroke centers from Dec 2015 to Dec 2018 (Registration: URL: http://www.chictr.org.cn/; Unique identifier: ChiCTR2000033211). Previously published work described PERSIST methodology in detail (10, 11).

In this study, we further excluded patients whose anesthetic method was not recorded specifically. The remaining patients were divided into two groups based on the anesthetic choice at the beginning of each EVT procedure: (1) patients who had endotracheal intubation along with general anesthesia (GA); (2) patients who had local anesthesia with or without sedation, as long as they had no endotracheal intubation (non-GA). Patients converted to GA during MT procedures were scored as non-GA according to the intention-to-treat principle. As-treated analysis considered the treatment actually received, which was sensitivity analysis. Patients in the non-GA group received a subcutaneous injection of Xylocaine and, if necessary, low-dose short-acting analgesics and/or sedatives. Patients in GA received analgesics and/or sedatives at higher doses at the discretion of anesthetists. In patients treated under GA, extubation was aimed for at the earliest time. The study was approved by the Ethics Committee of the First Affiliated Hospital of University of Science and Technology of China (USTC) in Hefei, China. Informed consent was waived by the Ethics Committee for this retrospective nature.

### Outcomes

The primary outcome measure was the favorable outcome, defined as a modified Rankin Scale (mRS) score of 0–3 at 90 days. Secondary outcomes included functional independence (90-day mRS score 0–2) and the rate of successful reperfusion (modified Thrombolysis in Cerebral Infarction score [mTICI], 2b−3) (12). Safety outcomes included all-cause mortality at 90 days, the occurrence of any procedural complication (dissection, perforation, and embolus in a new territory), and the rates of symptomatic intracranial hemorrhage (sICH). sICH was diagnosed if the newly observed ICH on imaging was related to any of the following conditions: (1) an NIHSS score that increased more than 4 points; (2) an NIHSS score that increased more than 2 points in a category; (3) deterioration led to hemicraniectomy, external ventricular drain placement, intubation, or other major medical interventions (13). All the neuroimaging data were sent to the core laboratory in the First Affiliated Hospital of USTC and were evaluated in a blinded manner by two experienced neuroradiologists. If there was any disagreement, the final assessment was confirmed on the basis of consensus.

### Statistical analysis

Continuous variables were described as the mean (SD) or median (IQR) as appropriate. Categorical variables were described as numbers (percentage). Normality of distributions was assessed using histograms and the Shapiro-Wilk test. To evaluate the magnitude of between-group differences for baseline characteristics, we calculated the absolute standardized difference, which interprets more than 10% as a meaningful difference (14). We compared the outcomes between the 2 groups after taking into account the potential confounding factors by using prespecified propensity score methods (PSM) (15).

The effects of the anesthetic approach were estimated by using propensity score matching as the primary analysis and by using the inverse probability of treatment weighting (IPTW) propensity score method (using stabilized inverse propensity score as weighty in regression models) as a secondary analysis. Patients in the GA group were matched 1:1 to patients in the non-GA group according to propensity score, using the greedy nearest neighbor matching algorithm, with a caliper of width equal to 0.2 of the standard deviation of the logit of the propensity score (16). A multivariable logistic regression model was used to compute the propensity score, with the anesthetic protocol as the dependent variable and all the baseline data in the table as covariates. Due to the lack of baseline data (range from 0 to 8%), the missing covariate values are processed through multiple imputation (chained equations with *m* = 5 imputations obtained) (17). The imputation procedure was performed under the missing at random assumption with a predictive mean matching method for continuous variables and logistic regression model for categorical variables. In each multiply imputed dataset, we calculated the propensity score and assembled a matched cohort to provide both matched and IPTW-propensity score-adjusted effect sizes, which were subsequently combined by using Rubin's rules (18).

Univariate analysis was performed using Student's *t*-test for continuous variables and χ^2^ or Fisher's exact test (for small cell size) for categorical variables. We compared the outcomes between groups by binary logistic regression analysis. Our initial analysis followed an intention-to-treat principle in which patients who converted from non-GA to GA during the procedure were included in the non-GA group. The as-treated analysis was also performed as a sensitive analysis. Statistical testing was conducted at the 2-tailed level of 0.05. All analyses were processed using SPSS version 26 (IBM Corp., Armonk, NY) and R version 4.0.5.

## Results

**Figure 1:**
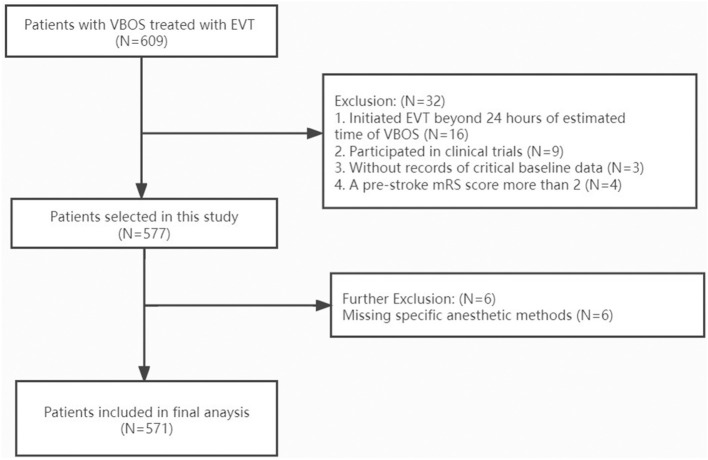
Flow chart of patient inclusion. VBAO indicates vertebrobasilar occlusion strokes; EVT, endovascular treatment; mRS, modified ranking scale.

The dataset of 609 patients with VBAO who received EVT during the study period included 571 patients [median age 64 (55–73) years, 71.5% male] who were ultimately eligible for analysis. The flow chart for the selection is presented in Figure 1. Of these, 451 patients underwent non-GA (80%) and 120 underwent GA (20%). The conversion from non-GA to GA occurred in 9/451 (2%) patients during the procedure because of severe movement or vomiting/aspiration. Both groups had similar medical histories, with the exception of atrial fibrillation and drinking history, which was more common in the GA group (Table 1). Patients in the GA group had a higher glucose level at admission, and more use of intravenous thrombolysis prior to EVT than patients in the non-GA group (Table 1). The admission systolic blood pressure, NIHSS score and GCS score were similar between the GA and non-GA groups, as well as the site of occlusion. The clinical characteristics and outcomes in the overall population without missing data imputation were shown in Supplementary Table 1.

**Table 1:** Clinical characteristics according to anesthetic approach in VBAO patients admitted for thrombectomy before and after propensity score matching.

**Characteristic**	**Before matching**			**After matching**		
	**GA (*****n*** = **120)**	**Non-GA (*****n*** = **451)**	**ASD, %**	**GA (*****n*** = **103)**	**Non-GA (*****n*** = **103)**	**ASD, %**
Age, median (IQR), year	66 (55–73)	64 (55–74)	3.4^*^	64 (54–73)	64 (54–72)	3.7^*^
Sex, male	92 (76.7)	316 (70.1)	15.0	76 (73.8)	77 (74.8)	2.3
**Medical history**						
Hypertension	82 (68.3)	304 (67.4)	2.0	70 (68.0)	71 (68.9)	1.9
Dyslipidemia	41 (34.2)	168 (37.3)	6.3	39 (37.9)	37 (35.9)	4.2
Atrial fibrillation	19 (15.8)	108 (23.9)	20.4	18 (17.5)	17 (16.5)	2.7
Diabetes	24 (20.0)	103 (22.8)	6.8	24 (23.3)	27 (26.2)	6.8
Coronary heart disease	10 (8.3)	46 (10.2)	6.6	7 (6.8)	9 (8.7)	7.1
Smoking	37 (30.8)	143 (31.7)	2.0	32 (31.1)	37 (35.9)	10.0
Drinking	18 (15.0)	94 (20.8)	15.2	16 (15.5)	18 (17.5)	5.4
**Clinical status**						
Admission SBP, mean (SD), mmHg	149 (23.2)	151 (25.3)	4.4	151 (21.5)	151 (31.0)	0.8
GCS score, median (IQR)	7 (6–11)	8 (6–12)	9.4^*^	8 (6–11)	7 (6–11)	5.6^*^
NIHSS score, median (IQR)	23 (14–28)	23 (14–30)	4.0^*^	23 (14–28)	22 (14–31)	2.3^*^
Glucose, median (IQR), mmol/L	8.2 (6.3–10.4)	7.2 (5.8–9.5)	13.9^*^	7.9 (6.3–10.4)	7.4 (5.8–10.3)	2.8^*^
**Site of occlusion**						
Basilar artery	86 (71.1)	342 (75.8)	9.3	75 (72.8)	71 (68.9)	8.6
**Treatment**						
IV thrombolyis	26 (21.7)	77 (17.1)	11.7	22 (21.4)	20 (19.4)	5.0

Values expressed as numbers (%) unless otherwise indicated. Values were calculated after handling missing data using multiple imputation procedure. ASD indicates absolute standardized difference; GA, general anesthesia; GCS, glasgow coma scale; IQR, interquartile range; IV, intravenous; NIHSS, national institutes of health stroke scale; non-GA, without general anesthesia; SD, standard deviation, and SBP, systolic blood pressure.

* Estimated using the rank-transformed data.

One hundred and three matched pairs were found in the primary analysis. The baseline characteristics according to the 2 study groups before and after PSM are shown in Table 1. Before matching, sex, atrial fibrillation history, drinking history, admission glucose levels and prior use of intravenous thrombolysis showed stronger differences (ASD > 10%). ASD decreased significantly after PSM with a maximum ASD of 2.3% for sex, 2.7% for atrial fibrillation history, 5.4% for drinking history, 2.8% for admission glucose levels, and 5% for prior use of intravenous thrombolysis (Table 1).

### Procedural-related outcomes and complications

The time from estimated occlusion to groin puncture between the GA and non-GA groups was not significant (*p* = 0.23); however, the time from groin puncture to reperfusion was 35 min longer in the GA group than in the non-GA group (*p* < 0.001) (Table 2). The rate of aspiration pneumonia was 78.6% in the GA group, which was significantly different from that in the non-GA group (63.1%) (*p* < 0.001). The remaining complications did not differ between patients who received GA and those who did not (Table 2). The rate of procedural complications occurred in 8 (6.7%) of 120 patients who had GA vs. 15 (11.1%) of 451 patients who had non-GA (*p* = 0.62).

**Table 2:** Procedural-related outcomes and complication according to anesthetic approach in VBAO patients admitted for thrombectomy before and after propensity score matching.

	**Before matching**			**After matching**		
	**GA (*****n*** = **120)**	**Non-GA (*****n*** = **451)**	***P*** **-value**	**GA (*****n*** = **103)**	**Non-GA (*****n*** = **103)**	***P*** **-value**
**Workflow**						
Estimated occlusion to groin puncture, median (IQR), hours	6.4 (4.0–8.5)	5.4 (3.8–8.7)	0.16	6.5 (4.0–8.8)	5.5 (3.9–8.3)	0.23
Onset to groin puncture, median (IQR), min	390 (252–500)	330 (233–513)	0.17	390 (255–500)	300 (215–480)	0.10
Groin puncture to reperfusion, median (IQR), min	140 (104–190)	103 (68–143)	< 0.001	140 (103–190)	105 (80–140)	<0.001
Procedural complication	8 (6.7)	50 (11.1)	0.16	8 (7.8)	10 (9.7)	0.62
Dissection	1 (0.8)	15 (3.3)	0.21	1 (1.0)	4 (3.9)	0.37
Perforation	3 (2.5)	11 (2.4)	1.00	3 (2.9)	2 (1.9)	1.00
Embolus in a new territory	4 (3.3)	24 (5.3)	0.48	4 (3.9)	4 (3.9)	1.00
**Complication**						
Pneumonia	95 (79.1)	268 (59.4)	< 0.001	81 (78.6)	65 (63.1)	<0.001
Cerebral hernia	18 (15.0)	43 (9.5)	0.09	13 (12.7)	7 (6.8)	0.17
Acute heart failure	22 (18.3)	65 (14.4)	0.29	18 (17.5)	10 (9.7)	0.10
Gastrointestinal bleeding	9 (7.5)	37 (8.2)	0.80	9 (8.7)	11 (10.7)	0.64

Values expressed as numbers (%) unless otherwise indicated. Values were calculated after handling missing data using multiple imputation procedure. GA, general anesthesia; IQR, interquartile range; non-GA, without general anesthesia.

### Primary and secondary outcomes

**Figure 2:**
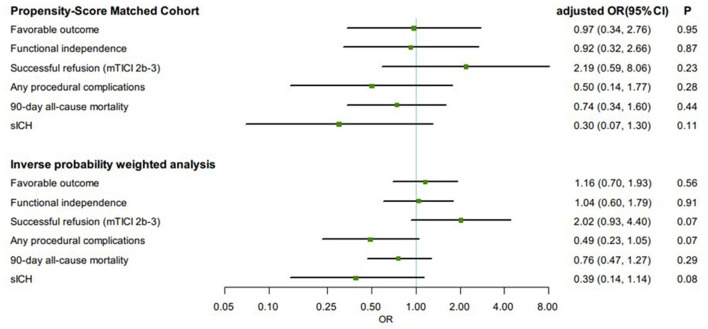
Comparisons in clinical and angiographic outcomes according to first-line anesthetic approach in patients treated with thrombectomy in matched and inverse probability of treatment weighting (IPTW) analyses. CI, confidence interval; mTICI, modified thrombolysis in cerebral infarction; OR, odds ratio; sICH, symptomatic intracranial hemorrhage. All regression analyses were adjusted for the following variables: age, sex, atrial fibrillation, smoking history, systolic blood pressure, glucose, site of occlusion, Glasgow Coma Scale score, baseline NIH Stroke Scale score, IV thrombolysis, pneumonia, time from estimated occlusion to groin puncture, door to groin puncture, and groin puncture to reperfusion.

In the propensity score matched cohort, the favorable outcome was not associated with any significant changes between the GA group and the non-GA group (aOR, 0.97, 95% CI 0.34–2.76, *p* = 0.95) (Figure 2). Similarly, the rate of functional independence (aOR, 0.92, 95% CI 0.32–2.66, *p* = 0.87) was not significantly different between the GA group and non-GA group, as well as the successful reperfusion (aOR, 2.19, 95% CI 0.59–8.06, *p* = 0.23). In the IPTW-propensity score-adjusted cohort, similar results were found in favorable outcome, functional independence and successful reperfusion (Figure 2). With respect to the safety outcomes, we found no significant differences in the PSM cohort, which showed the same outcomes in the IPTW-propensity cohorts (Figure 2). The sensitivity analysis restricted to the as-treated sample provided similar results across all studied outcomes in the PSM cohort as well as in the IPTW-propensity cohorts (Supplementary Tables 2, 3 and Supplementary Figure 1).

### Subgroup analysis

**Figure 3:**
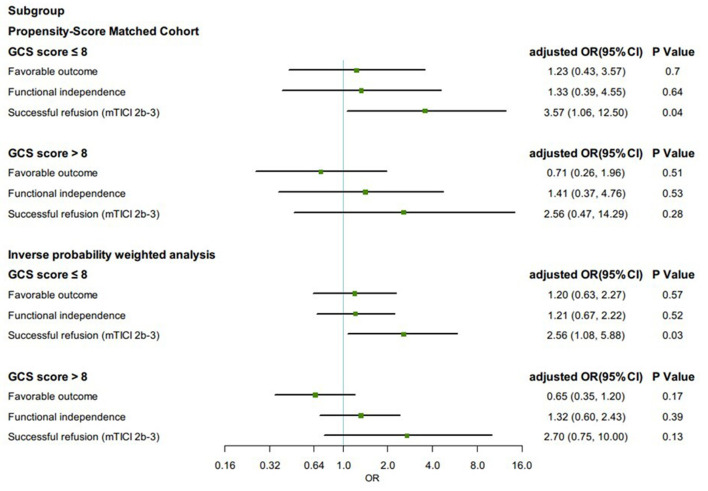
Subgroup analysis in clinical and angiographic outcomes according to first-line anesthetic approach in patients treated with thrombectomy in matched and inverse probability of treatment weighting (IPTW) analyses. CI, confidence interval; mTICI, modified thrombolysis in cerebral infarction; OR, odds ratio; sICH, symptomatic intracranial hemorrhage. All regression analyses were adjusted for the following variables: age, sex, atrial fibrillation, smoking history, systolic blood pressure, glucose, site of occlusion, baseline NIH Stroke Scale score, IV thrombolysis, pneumonia, time from estimated occlusion to groin puncture, door to groin puncture, and groin puncture to reperfusion.

Propensity score-matched patients in each group were divided into subgroups by Glasgow Coma Scale (GCS) score (≤8 or >8). Among patients with GCS score ≤8, the proportion of successful reperfusion was significantly higher in the GA group (88.5%) than in the non-GA group (79.6%) (aOR, 3.57, 95% CI 1.06–12.50, *p* = 0.04). The effect on favorable outcome (aOR, 1.23, 95% CI 0.43–3.57, *p* = 0.70), functional independence (aOR, 1.33, 95% CI 0.39–4.55, *p* = 0.64), and all safety outcomes remained no differences. In IPTW-propensity score-adjusted cohort, similar results were presented (Figure 3).

## Discussion

After adjustment for baseline characteristics, our study showed that VBAO patients who underwent GA achieved similar rates of primary and secondary outcomes as those who had non-GA, meanwhile, without an increased risk of symptomatic intracranial hemorrhage or mortality. The results were similar using both intention-to-treat and as-treated analysis. Additionally, subgroup analysis based on GCS score revealed that in VBAO patients with a lower presenting GCS score (≤8), GA could yield a higher proportion of successful reperfusion.

Most early observational studies demonstrated that patients with acute ischemic stroke undergoing EVT appeared to show worse neurological outcomes and higher mortality when treated with GA compared with non-GA (19, 20). Delay in treatment initiation (due to the time required for GA induction, emergency endotracheal intubation, and an available experienced anesthesiologist) has been speculated to be a reasonable explanation as to why GA may be associated with poorer neurological outcomes after EVT. The highly effective reperfusion using multiple endovascular devices (HERMES) collaboration (21), for example, the time interval between randomization and reperfusion was 20 min later in patients who had GA vs. patients who had non-GA during EVT in anterior circulation ischemic stroke. However, in the present study, puncture to reperfusion times were significantly longer in the GA group that did not confer a disadvantage to patients with VBAO compared to non-GA. A recent meta-analysis showed that in spite of the longer onset-to-EVT and onset-to-groin puncture times in VBAO, favorable functional outcome at 90 days in VBAO was comparably no difference just as in anterior circulation large vessel occlusion during EVT (22). This could be supported by the hypothesis that benefit of recanalization is less time-dependent in VBAO than in anterior circulation large vessel occlusion due to the anatomical vascular layout of the brainstem being different from that in usual anterior circulation stroke (23).

Compared with anterior circulation stroke, VBAO has its own characteristics. Patients with VBAO are more likely to have consciousness disorders, or even remain in a deep coma. Therefore, local anesthesia with or without conscious sedation is safe and effective for these patients, especially for operations that can be completed quickly. Most patients with consciousness disorders are prone to restless, accompanied by irregular breathing patterns, hypoxemia, vomiting and aspiration, especially patients with difficult vascular approach and predicted long operation time, so general anesthesia remains widely used for mechanical thrombectomy treatment of acute ischemic stroke. General anesthesia may provide optimal conditions for procedural operations, fewer technical failures and complications occur and higher recanalization rates are achieved, resulting in better clinical outcomes (24). However, no studies have certified that conscious sedation is associated with higher rates of wire perforation, dissection, or intracranial hemorrhage than general anesthesia (20). Additionally, general anesthesia is more frequently associated with hemodynamic instability, such as intraoperative hypotension, which may lead to worse outcomes. Therefore, it is likely that standard circulation management is essential in reducing the negative effects of hemodynamic fluctuations. To our knowledge, the first RCT to compare anesthetic management in patients with VBAO during EVT found that there were not notably different in rates of 90-day favorable outcomes, mortality successful reperfusion, intraoperative hypotension, or perioperative changes in systolic blood pressure between conscious sedation and GA (8). Our study found similar clinical outcomes and safety outcomes between the GA and non-GA groups during vertebrobasilar stroke endovascular therapy, in line with several RCTs and observational studies (4–6, 8, 25, 26). However, a systematic review and meta-analysis found that non-GA was associated with better outcomes than GA in patients with acute posterior circulation stroke undergoing EVT (27). These findings were inconsistent with ours, which might be explained by the differences in baseline patient characteristics in the meta-analysis, such as stroke severity.

VBAO may lead to bulbar palsy and/or consciousness impairment, which increases periprocedural complications; hence, selection bias was prone to general anesthesia for patients with more severe illness. A lower presenting Glasgow Coma Scale score (≤8) was predictive of poor patient outcome in endovascular treatment for acute posterior large-vessel occlusion (28, 29). According to subgroup analysis, in patients with a GCS score ≤8, a significantly higher rate of successful reperfusion was observed in the GA group. However, the difference in recanalization rate of this size was not sufficient to explain the other outcomes that we observed in our study. Due to the small sample size of the subgroup analysis, the width of 95% confidence intervals was comparatively large. Interestingly, a pilot trial of 43 patients with acute anterior circulation ischemic stroke who underwent EVT found results similar to us, which showed that the rate of successful reperfusion (mTICI score 2b-3) was greater in the patients allocated to receive general anesthesia, and showed no difference in NIHSS scores at 24 h or 7 days or mRS scores at 30 days (30). For patients with poorer clinical presentation and more severe stroke, GA is perhaps more favorable than non-GA in rates of successful reperfusion.

We acknowledge that our study has several limitations. First, it is not clear whether the better functional outcomes in the non-GA group are merely related to non-intubated anesthesia. Second, as a retrospective study, we took advantage of propensity score to adjust for potential confounders between groups. However, the results could have been confounded by variables not accounted for in the propensity model. Third, the anesthesiologist and operator adopted the most appropriate anesthesia strategy for each patient, based on their experience combined with the patient's situation before operation, which lacked a unified agreement. In addition, we did not investigate potentially important procedural factors that could have affected our findings, such as periprocedural blood pressure fluctuations. Finally, we could not avoid the bias caused by multiple imputations that were used to deal with missing data.

## Conclusions

Our study suggests that GA appears to be as safe and effective as non-GA during EVT of VBAO. In addition, for patients with GCS score ≤8, we may give priority to general anesthesia. Future prospective studies are warranted, at least to extend our understanding of the effect of the anesthesia strategy and help determine the best anesthetic modality during EVT of VBAO.

## Data availability statement

The raw data supporting the conclusions of this article will be made available by the authors, without undue reservation.

## Ethics statement

The studies involving human participants were reviewed and approved by the Ethics Committee of the First Affiliated Hospital of University of Science and Technology of China (USTC) in Hefei, China. Written informed consent for participation was not required for this study in accordance with the national legislation and the institutional requirements.

## Author contributions

YL, PZ, WS, ZC, and ML contributed to the study concept and design. PX, JW, LX, ZD, DeW, CZ, and TY contributed to acquisition and analysis of data. DL, CL, and DiW contributed to image review and drafting figures of the manuscript. YL, WS, ZC, and ML contributed to drafting the text. All authors contributed to the article and approved the submitted version.
