# Supplementary material for: General anesthesia vs. non-general anesthesia for vertebrobasilar stroke endovascular therapy
Source: Front Neurol. 2023 Feb 2;14:1104487. doi: 10.3389/fneur.2023.1104487 (PMC9932259; doi:10.3389/fneur.2023.1104487)
Supplement: Supplementary file 1 [file Data_Sheet_1.docx]

Supplemental Table I. Clinical Characteristics and Outcomes According to Anesthetic Approach in the Overall Population Without Missing Data Imputation

| Characteristic | GA (n =120) | Non-GA (n =451) | P value |
| --- | --- | --- | --- |
| Age, median (IQR), y | 66 (55-73) | 64 (55-74) | 0.92 |
| Sex, male | 92 (76.7) | 316 (70.1) | 0.16 |
| Medical history |  |  |  |
| Hypertension | 82 (68.3) | 304 (67.4) | 0.85 |
| Dyslipidemia | 41 (34.2) | 168 (37.3) | 0.53 |
| Atrial fibrillation | 19 (15.8) | 108 (23.9) | 0.06 |
| Diabetes | 24 (20.0) | 103 (22.8) | 0.51 |
| Coronary Heart Disease | 9 (7.5) | 47 (10.4) | 0.34 |
| Smoking | 37 (30.8) | 143 (31.8) | 0.84 |
| Drinking | 18 (15.0) | 94 (20.9) | 0.15 |
| Clinical status |  |  |  |
| Admission SBP, mean (SD), mmHg | 149 (23.2) | 151 (25.3) | 0.95 |
| GCS score, median (IQR) | 7 (6-11) | 8 (6-12) | 0.44 |
| NIHSS score, median (IQR) | 23 (14-28) | 23 (14-30) | 0.65 |
| Glucose, median (IQR), mmol/L | 7.9 (6.3-10.1) | 7.1 (5.8-9.1) | 0.15 |
| Site of occlusion |  |  |  |
| Basilar artery | 86 (71.7) | 342 (75.8) | 0.35 |
| Treatment |  |  |  |
| IV thrombolyis | 26 (21.7) | 77 (17.1) | 0.25 |
| Workflow |  |  |  |
| Estimated occlusion to groin puncture, median (IQR), hours | 6.4 (4.0-8.5) | 5.4 (3.8-8.7) | 0.16 |
| Onset to groin puncture, median (IQR), min | 390 (252-500) | 330 (234-514) | 0.18 |
| Groin puncture to reperfusion, median (IQR), min | 146 (105-192.5) | 101.5 (66.5-140) | <0.001 |
| Procedural complication |  |  |  |
| Dissection | 1 (0.8) | 15 (3.3) | 0.21 |
| Perforation | 3 (2.5) | 11 (2.4) | 1.00 |
| Embolus in a new territory | 4 (3.3) | 24 (5.3) | 0.48 |
| Complication |  |  |  |
| Pneumonia | 95 (79.2) | 268 (59.6) | <0.001 |
| Cerebral hernia | 15 (12.8) | 43 (9.6) | 0.30 |
| Acute heart failure | 22 (18.3) | 65 (14.4) | 0.29 |
| Gastrointestinal bleeding | 9 (7.5) | 37 (8.2) | 0.80 |
| Outcomes |  |  |  |
| Successful reperfusion (mTICI2b-3) | 109 (90.8) | 381 (84.5) | 0.08 |
| sICH | 4 (3.3) | 34 (7.5) | 0.07 |
| 90-d mRS 0-2 | 33 (27.5) | 141 (31.3) | 0.43 |
| 90-d mRS 0-3 | 42 (35.0) | 177 (39.2) | 0.40 |
| 90-d mortality | 43 (35.8) | 171 (37.9) | 0.68 |

Values expressed as numbers (%) unless otherwise indicated. Values were calculated after handling missing data using multiple imputation procedure. ASD indicates absolute standardized difference; GA, general anesthesia; GCS, Glasgow Coma Scale; IQR, interquartile range; IV, intravenous; NIHSS, National Institutes of Health Stroke Scale; non-GA, without general anesthesia; sICH, symptomatic intracranial hemorrhage; SD, standard deviation, and SBP, systolic blood pressure.

Supplemental Table II. Clinical Characteristics According to Anesthetic Approach in VBAO Patients Admitted for Thrombectomy Before and After Propensity Score Matching, in as-treated sample.

|  | Before Matching | | | After Matching | | |
| --- | --- | --- | --- | --- | --- | --- |
| Characteristic | GA (n =129) | Non-GA (n =442) | ASD, % | GA (n =106) | Non-GA (n = 106) | ASD, % |
| Age, median (IQR), y | 66 (55-73) | 64 (55-74) | 2.2^*^ | 66 (55-72) | 64 (54-74) | 1.6^*^ |
| Sex, male | 97 (75.2) | 311 (70.4) | 10.8 | 77 (72.6) | 81 (76.4) | 9.9 |
| Medical history |  |  |  |  |  |  |
| Hypertension | 88 (68.2) | 298 (67.4) | 1.7 | 76 (71.7) | 73 (68.8) | 6.4 |
| Dyslipidemia | 46 (35.7) | 163 (36.9) | 2.5 | 39 (36.8) | 38 (35.8) | 2.1 |
| Atrial fibrillation | 21 (16.3) | 106 (24.0) | 19.3 | 18 (17.0) | 15 (14.2) | 7.7 |
| Diabetes | 26 (20.2) | 101 (22.9) | 6.6 | 24 (22.6) | 27 (25.5) | 6.8 |
| Coronary Heart Disease | 10 (7.8) | 46 (10.4) | 9.1 | 10 (9.4) | 10 (9.4) | 0.0 |
| Smoking | 38 (29.5) | 142 (32.1) | 5.6 | 31 (29.2) | 29 (27.4) | 4.0 |
| Drinking | 18 (14.0) | 94 (21.3) | 19.2 | 15 (14.2) | 16 (15.1) | 2.6 |
| Clinical status |  |  |  |  |  |  |
| Admission SBP, mean (SD), mmHg | 150 (23.7) | 151 (25.2) | 5.0 | 149 (23.0) | 150 (26.0) | 4.1 |
| GCS score, median (IQR) | 8 (6-11) | 8 (6-12) | 5.0^*^ | 8 (6-11) | 8 (6-12) | 2.2^*^ |
| NIHSS score, median (IQR) | 22 (13-28) | 23 (14-30) | 9.1^*^ | 22 (14-28) | 22 (13-28) | 6.5^*^ |
| Glucose, median (IQR),  mmol/L | 7.9 (6.3-10.3) | 7.2 (5.8-9.5) | 9.6^*^ | 8.0 (6.3-10.3) | 7.4 (6.1-10.5) | 9.8^*^ |
| Site of occlusion |  |  |  |  |  |  |
| Basilar artery | 90 (69.8) | 338 (76.5) | 15.2 | 79 (74.5) | 82 (77.4) | 6.8 |
| Treatment |  |  |  |  |  |  |
| IV thrombolyis | 29 (22.5) | 74 (16.8) | 14.4 | 22 (20.7) | 20 (18.9) | 4.5 |

Values expressed as numbers (%) unless otherwise indicated. Values were calculated after handling missing data using multiple imputation procedure. ASD indicates absolute standardized difference; GA, general anesthesia; GCS, Glasgow Coma Scale; IQR, interquartile range; IV, intravenous; NIHSS, National Institutes of Health Stroke Scale; non-GA, without general anesthesia; SD, standard deviation, and SBP, systolic blood pressure.

*Estimated using the rank-transformed data.

Supplemental Table III. Procedural-related Outcomes and Complication According to Anesthetic Approach in VBAO Patients Admitted for Thrombectomy Before and After Propensity Score Matching, in as-treated sample.

|  | Before Matching | | | After Matching | | |
| --- | --- | --- | --- | --- | --- | --- |
|  | GA (n =129) | Non-GA (n =442) | P value | GA (n =106) | Non-GA (n = 106) | P value |
| Workflow |  |  |  |  |  |  |
| Estimated occlusion to groin puncture, median (IQR), hours | 6.4 (4.0-8.7) | 5.4 (3.8-8.6) | 0.23 | 6.5 (4.1-8.7) | 5.5 (3.6-8.0) | 0.10 |
| Onset to groin puncture,  median (IQR), min | 390 (250-500) | 330 (235-510) | 0.16 | 390 (254-500) | 325 (221-444) | 0.05 |
| Groin puncture to  reperfusion,  median (IQR), min | 140 (105-188) | 102 (67-144) | <0.001 | 140 (106-194) | 110 (80-150) | <0.001 |
| Procedural complication | 9 (6.7) | 49 (11.1) | 0.17 | 9 (8.5) | 13 (12.3) | 0.37 |
| Dissection | 2 (2) | 14 (3.4) | 0.26 | 2 (1.9) | 4 (3.8) | 0.34 |
| Perforation | 3 (2.3) | 11 (2.5) | 0.61 | 3 (2.8) | 3 (2.8) | 0.66 |
| Embolus in a new  Territory | 4 (3.1) | 24 (5.4) | 0.20 | 4 (3.8) | 6 (5.7) | 0.37 |
| Complication |  |  |  |  |  |  |
| Pneumonia | 103 (79.8) | 260 (58.8) | <0.001 | 81 (78.6) | 65 (63.1) | 0.004 |

Values expressed as numbers (%) unless otherwise indicated. Values were calculated after handling missing data using multiple imputation procedure. GA, general anesthesia; IQR, interquartile range; non-GA, without general anesthesia.


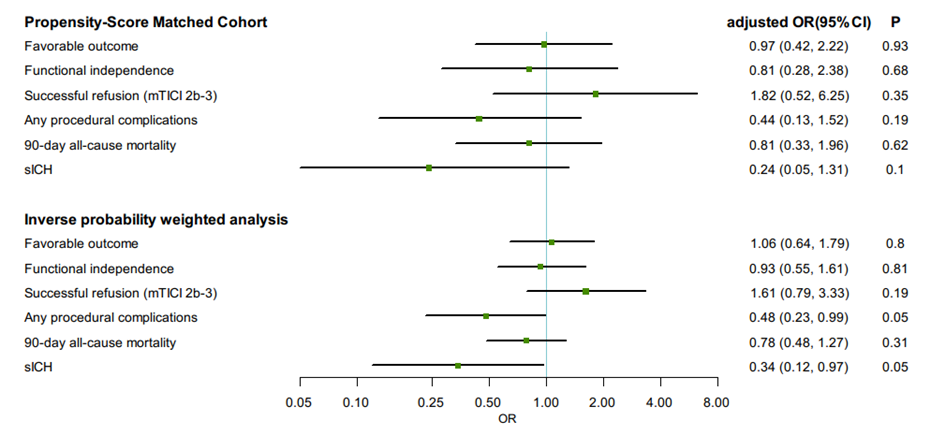


Supplemental Figure I. Comparisons in clinical and angiographic outcomes according to first-line anesthetic approach in patients treated with thrombectomy in matched and inverse probability of treatment weighting (IPTW) analyses, in as-treated sample. Abbreviations: CI, confidence interval; mTICI, modified Thrombolysis in Cerebral Infarction; OR, odds ratio; sICH, symptomatic intracranial hemorrhage. All regression analyses were adjusted for the following variables: age, sex, atrial fibrillation, smoking history, systolic blood pressure, glucose, site of occlusion, Glasgow Coma Scale score, baseline NIH Stroke Scale score, IV thrombolysis, pneumonia, time from estimated occlusion to groin puncture, door to groin puncture, groin puncture to reperfusion.
